# Application of the International Endometrial Tumor Analysis (IETA) in Ultrasound Evaluation of Abnormal Uterine Bleeding

**DOI:** 10.7759/cureus.66560

**Published:** 2024-08-10

**Authors:** Yashraj Patil, Aryaman Dhande, Sanjay M Khaladkar, Prajakta P Kirdat Patil

**Affiliations:** 1 Radiodiagnosis, Dr. D. Y. Patil Medical College, Hospital & Research Centre, Pune, IND

**Keywords:** ieta, endometrial pathology, international endometrial tumor analysis, ultrasonography, abnormal uterine bleeding

## Abstract

Introduction: Abnormal uterine bleeding (AUB) affects a significant proportion of women, particularly around the ages of menarche and menopause. While ultrasonography is a primary diagnostic tool for AUB, techniques like the International Endometrial Tumor Analysis (IETA) scoring system have enhanced diagnostic accuracy for endometrial abnormalities. IETA provides a standardized approach to evaluating endometrial features, which aids in distinguishing benign from malignant lesions.

Methods: This study applied the IETA scoring system to the ultrasound evaluation of 50 women presenting with AUB. The study assessed various endometrial characteristics, including thickness, echogenicity, midline appearance, junction regularity, and vascular patterns. Data were analyzed to correlate IETA scores with histopathological findings and to compare the ultrasound features of benign and malignant lesions.

Results: The study found that non-uniform endometrial characteristics and irregular midline appearances were more common in malignant lesions. Specifically, interrupted or irregular endometrial-myometrial junctions, absence of the bright edge, higher color scores, and complex vascular patterns were significantly associated with malignancy. Mean endometrial thickness was notably higher in malignant cases compared to benign ones, with a statistically significant difference. The most frequent IETA scores were 7, 12, and 13.

Conclusion: Integrating the IETA scoring system into ultrasound evaluation enhances the detection of endometrial abnormalities, improving the differentiation between benign and malignant lesions. This approach provides a reliable framework for diagnosing and managing AUB, potentially reducing the need for invasive procedures and facilitating better clinical decision-making.

## Introduction

Abnormal uterine bleeding (AUB) is a prevalent gynecological concern affecting women of reproductive age worldwide, with estimates ranging widely from 3% to 30%. This condition, characterized by irregularities in menstrual bleeding patterns beyond heavy menstrual bleeding (HMB), encompasses a significant portion of gynecological consultations, particularly during menarche and peri-menopause [1].

Ultrasonography has emerged as an indispensable tool in the diagnostic arsenal for AUB due to its non-invasive nature, widespread availability, and cost-effectiveness [2]. The advent of techniques such as the International Endometrial Tumor Analysis (IETA) has notably advanced the accuracy and precision in identifying the underlying causes of AUB [3]. Established in 2008, the IETA group has standardized guidelines for evaluating the sonographic features of the endometrium and uterine cavity [4]. These guidelines employ gray-scale sonography, color flow imaging, and sonohysterography to systematically assess endometrial characteristics, offering specific terms, definitions, and measurements for enhanced diagnostic clarity [4]. Central to the IETA framework is its straightforward scoring system, where a threshold score of ≥6.5 points indicates significant diagnostic utility in distinguishing benign from malignant uterine and endometrial lesions [5]. Integration of IETA into routine ultrasound examinations for AUB not only improves diagnostic accuracy by providing uniform criteria for detecting endometrial abnormalities but also facilitates more informed clinical decision-making, reduces reliance on invasive procedures, and promotes consistency in reporting and interpretation [5,6]. The system evaluates specific characteristics such as endometrial thickness, echogenicity patterns, midline appearance, junction regularity, presence of a bright edge, intracavitary fluid types, color score, and vascular patterns. Each characteristic is categorized into defined ranges or patterns, with higher scores indicating more significant or abnormal findings [5].

Gray-scale sonography assesses endometrial thickness, echogenicity, and focal lesions, with normal endometrium presenting a homogeneous pattern while conditions like polyps, hyperplasia, or cancer manifesting as heterogeneous or thickened endometrium. Doppler ultrasound evaluates endometrial vascularity, where increased vascularity may indicate malignancy or hyperplasia, while polyps typically exhibit a single feeding vessel. Sonohysterography involves saline or gel instillation into the uterine cavity to enhance visualization, aiding in the detection of intracavitary lesions such as polyps or submucosal fibroids, and distinguishing focal from diffuse endometrial abnormalities [7].

A comprehensive assessment of AUB often involves both transabdominal and transvaginal ultrasounds concurrently, with transabdominal ultrasound providing a panoramic view of pelvic organs and transvaginal ultrasound offering detailed views of the uterus and fibroids. In selected cases, additional imaging modalities such as magnetic resonance imaging (MRI) offer superior soft-tissue contrast, crucial for accurately distinguishing fibroids from other myometrial pathologies. Despite the recognized value of ultrasound in gynecology, standardized methodologies for comprehensive examination to improve diagnostic accuracy and treatment planning remain underdeveloped. Addressing this gap, the present study aims to integrate advanced ultrasound techniques within a robust diagnostic framework for AUB, with a specific focus on describing myometrial pathologies and distinguishing between benign and malignant endometrial conditions using ultrasound guided by IETA.

## Materials and methods

Study setting

An observational descriptive study was conducted in the Department of Radiodiagnosis at Dr. D. Y. Patil Medical College, Hospital & Research Centre, Pimpri, Pune, from August 2022 to July 2024. The study spanned two years and involved 50 participants presenting with AUB and suspected endometrial pathologies. The sample size of 50 was chosen based on feasibility, balancing the need for meaningful analysis with practical constraints. Conducted over two years at a single institution, this size allows for a thorough evaluation of IETA's utility while managing resources effectively. Although not large enough for extensive generalization, it provides sufficient data for preliminary insights and can guide future research.

Participant selection

This study included women of pre- and post-menopausal age who presented with clinically symptomatic AUB, aiming to investigate endometrial pathologies comprehensively. Women with a history of hysterectomy and those experiencing AUB due to general factors such as medications, hormonal influences, systemic diseases, or local factors, including lesions of the vulva, vagina, or cervix, as well as obstetric causes, were excluded. By narrowing the inclusion criteria to symptomatic AUB cases, this research aimed to provide a detailed examination specifically targeting endometrial abnormalities, utilizing advanced ultrasound techniques guided by the IETA framework.

Data collection

Demographic details, including age, clinical history, and specific characteristics of AUB, were meticulously recorded for all participants. Screening involved the use of the Samsung HS70A with PRIME technology (Samsung, Suwon, South Korea), with each participant undergoing both transabdominal ultrasonography (TAUS) and transvaginal ultrasonography (TVS). TVS provided a detailed assessment within a limited depth, utilizing dynamic 2D scans to evaluate uterine mobility and identify site-specific tenderness. Sonographic cross-sections enabled visualization of arcuate venous and arterial vessels near the outer myometrial border. TAUS complemented TVS by offering broader pelvic imaging capabilities. Key evaluation parameters included endometrial thickness, echogenicity, midline appearance, myometrial junction appearance, presence of a bright edge, characteristics of intracavitary fluid, color score, vascular pattern, and IETA scores. The IETA classification system, employed during transvaginal ultrasound, standardized the evaluation of endometrial pathologies based on echogenicity, thickness, and structural abnormalities. This systematic approach facilitated differentiation between normal endometrial patterns and pathological conditions such as hyperplasia or cancer. Histopathological examination was also conducted to confirm and correlate the USG findings with definitive diagnoses.

Scoring system for ultrasound characteristics of the endometrium

**Figure 1:**
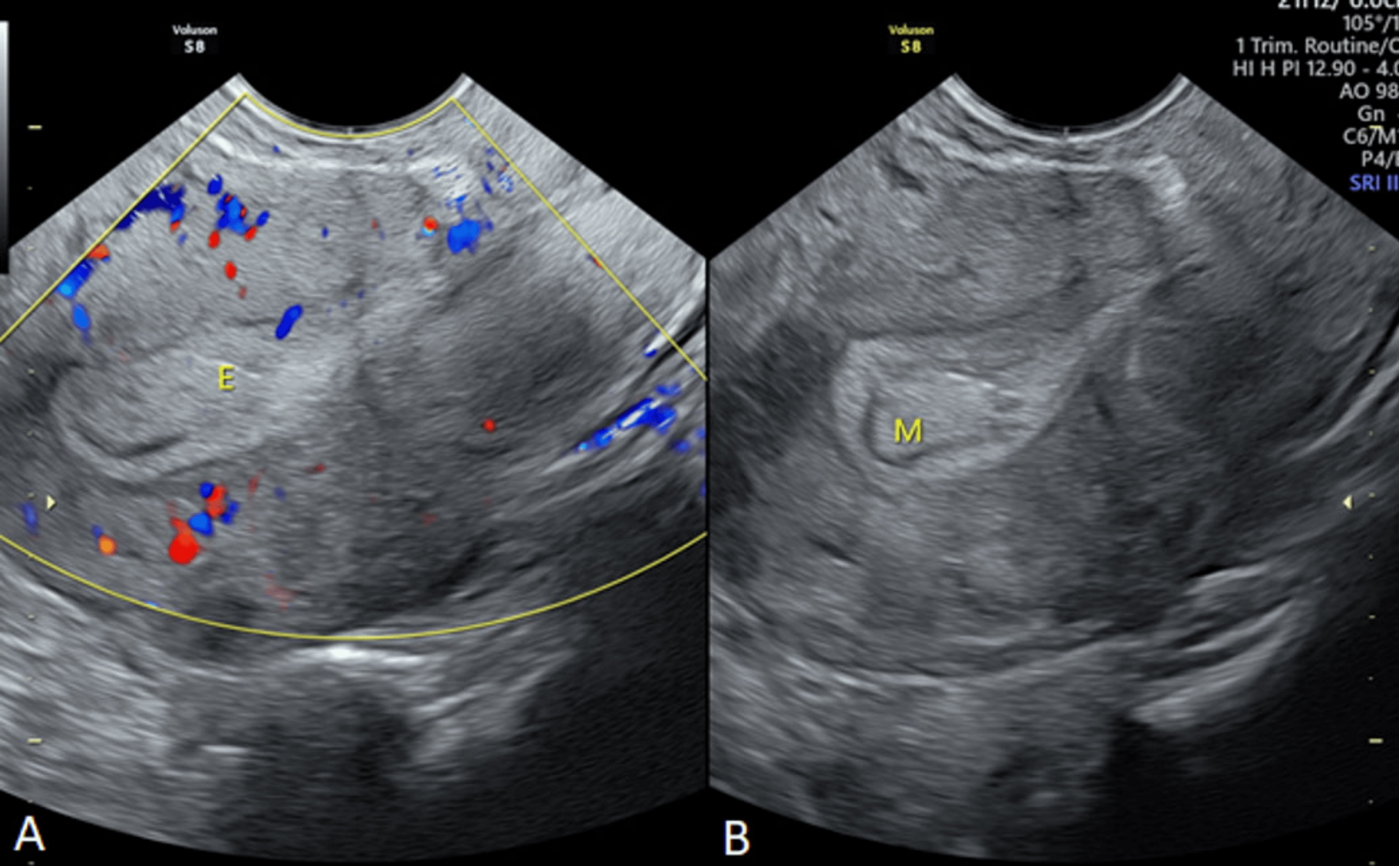
Transvaginal ultrasonography (TVS). (A) Color Doppler flow image of the endometrial lesion. (B) Grayscale image of the endometrial lesion in a female, 27 years old, with abnormal menstruation for more than two months. The postoperative pathology was an endometrial polyp, and the total score of the International Endometrial Tumor Analysis (IETA) ultrasound characteristics simple scoring method was 3 points (endometrial thickness 12 mm: 0 points; heterogeneous without cysts: 1 point; regular endometrial–myometrial junction: 0 points; having “Bright edge” sign: 0 points; color score: sparse vascularity: 2 points; single vessel (without branching): 0 points).

A score of 0 points indicated features such as an endometrial thickness of ≤12.0 mm (premenopause) or ≤5.0 mm (postmenopause), uniform endometrial echogenicity, a linear, nonlinear, irregular, or undefined endometrial midline appearance, a regular endometrial-myometrial junction (EMJ), the presence of a "Bright Edge," absence of intracavitary fluid, and specific vascular patterns like no flow or a single vessel with or without branching, scattered vessels, or circular vessels. A score of 1 point represented slightly increased endometrial thickness (12.1-15.0 mm in premenopausal and 5.1-10.0 mm in postmenopausal women), non-uniform echogenicity (including irregular cysts), an irregular EMJ, absence of a "Bright Edge," anechogenic intracavitary fluid, no color or vascularity on the color score, and multiple vessels with a focal origin. Score 2 indicated findings such as endometrial thickness (15.1-20.0 mm in premenopausal and 10.1-15.0 mm in postmenopausal women), non-uniform echogenicity of the endometrium, interrupted EMJ, presence of intracavitary fluid with a "ground glass" appearance, minimal color/vascularity on the color score, and multiple vessels with multifocal origin. Score 3 represented findings such as a significant increase in endometrial thickness (>20.1 mm in premenopausal and >15.1 mm in postmenopausal women), undefined EMJ, intracavitary fluid with "mixed" echogenicity, and moderate vascularity on the color score. Score 4 was assigned to a color score with abundant vascularity. An example of the scoring system has been explained in Figure 1.

To maintain high standards in ultrasound imaging, operators received comprehensive training to ensure consistent technique and diagnostic accuracy. The ultrasound equipment was calibrated regularly following manufacturer guidelines to uphold image quality. Standardized imaging protocols were used to reduce variability, including uniform settings for endometrial evaluations. Additionally, images were routinely reviewed and verified by experienced radiologists to confirm the reliability of the results.

Statistical analysis

Data entry was done in Microsoft Excel (Microsoft Corporation, Redmond, WA) and analyzed using SPSS (V 23.0, IBM Corp., Armonk, NY). All patient information and study data were stored in a secure, encrypted database, accessible only to authorized personnel. Descriptive statistics (mean, standard deviation, range, frequencies, and percentages) summarized demographic and clinical data. Correlation analysis between USG findings and histopathological diagnoses was performed using chi-squared tests, likelihood ratios, and independent sample t-tests. A p-value of <0.05 was considered statistically significant.

## Results

The age of the participants varied from 20 to 65 years, with a range of 45 years. The mean age of the sample was 38.48 years. The standard deviation was 11.33, indicating the variability of ages around the mean. Uniform lesions included hyperechogenic (5, 10.0%), hypoechoic (4, 8.0%), and isoechoic (3, 6.0%) types. Non-uniform lesions were predominantly heterogeneous with irregular cysts (20, 40.0%), followed by heterogeneous without cysts (9, 18.0%), and homogeneous with irregular cysts (9, 18.0%). This variability in echogenicity types suggests diverse histopathological features among endometrial lesions, influencing diagnostic considerations and patient management strategies. This variability in echogenicity types suggests diverse histopathological features among endometrial lesions, influencing diagnostic considerations and patient management strategies (Table 1).

**Table 1:** Distribution of study participants based on the type of endometrial echogenicity.

Type of lesions		Frequency	Percent
Uniform lesions	Hyperechogenic	5	10.0
	Hypoechoic	4	8.0
	Isoechoic	3	6.0
	Total	12	100.0
Non-uniform lesions	Heterogeneous with irregular cysts	20	40.0
	Heterogeneous without cysts	9	18.0
	Homogeneous with irregular cysts	9	18.0
	Total	38	100.0

On histopathological examination, benign lesions were predominantly represented by endometrial polyps, accounting for 14 (87.5%) of the benign cases. Endometrial simple hyperplasia comprised the remaining two cases (12.5%). Among malignant lesions, endometrial hyperplasia with atypia was the most prevalent, representing 15 (44.1%) cases. Endometrioid carcinoma was the second most common, comprising 13 (38.2%), endometrial complex hyperplasia accounted for four (11.8%), and the International Federation of Gynecology and Obstetrics (FIGO) stage IA was the least common at two (5.9%) out of the total malignant cases (Table 2).

**Table 2:** Distribution of the study participants based on the type of endometrial lesions. FIGO: International Federation of Gynecology and Obstetrics staging of endometrial cancers.

Type of endometrial lesion		Frequency	Percent
Benign lesions	Endometrial polyp	14	87.5
	Endometrial simple hyperplasia	2	12.5
	Total	16	100.0
Malignant lesions	Endometrial complex hyperplasia	4	11.8
	Endometrial hyperplasia with atypia	15	44.1
	Endometrioid carcinoma	13	38.2
	FIGO stage IA	2	5.9
	Total	34	100.0

**Figure 2:**
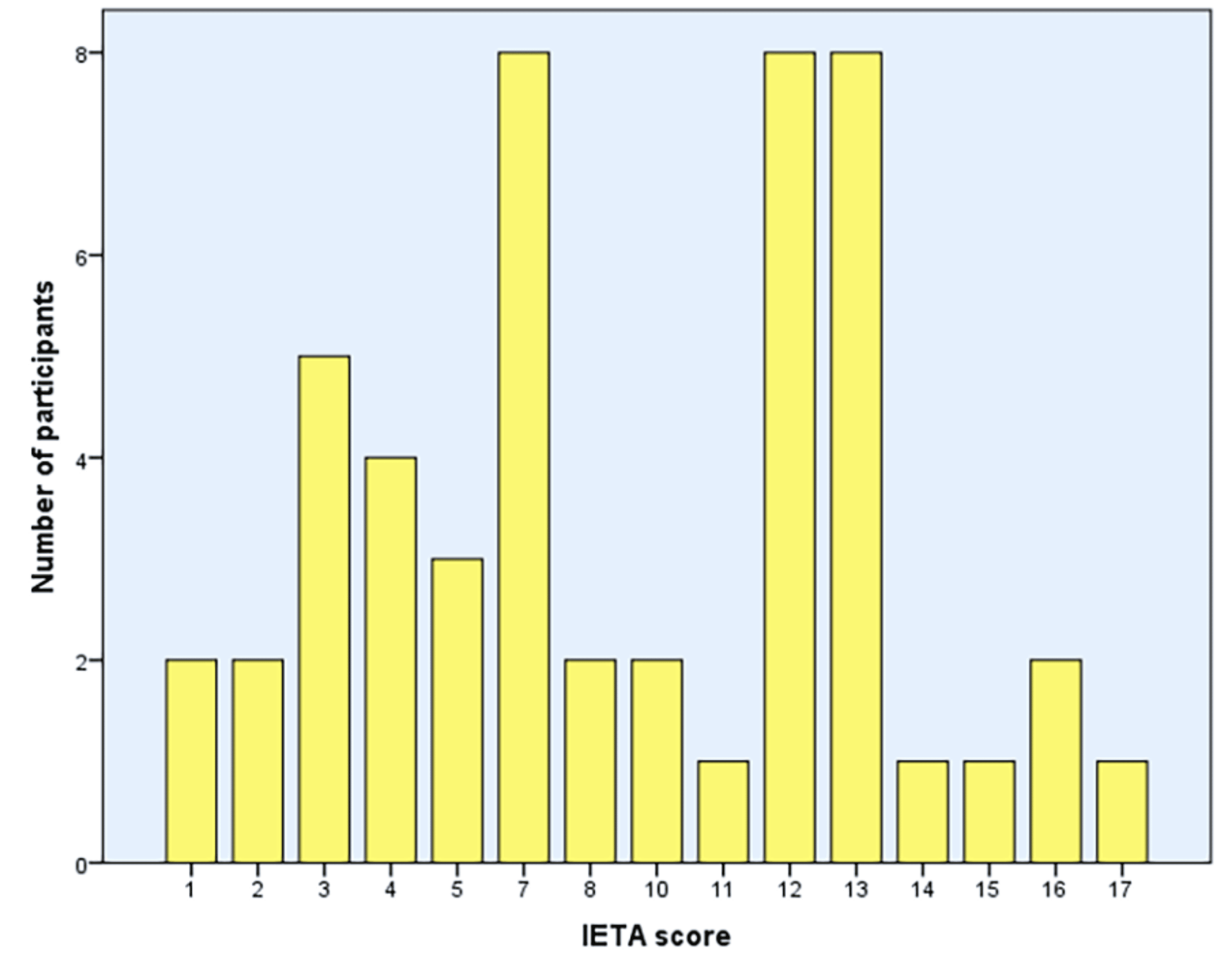
Bar chart showing the distribution of study participants on the basis of the IETA scores. IETA: International Endometrial Tumor Analysis.

Distribution of the IETA scores among participants showed scores of 7, 12, and 13 were among the highest number of participants (Figure 2).

A comparative analysis of various ultrasound characteristics between benign and malignant lesions, including uniformity, midline appearance, junction type, presence of a bright edge, intracavitary fluid types, color score, vascular patterns, ultrasound diagnosis, and endometrial thickness was done. For uniformity, non-uniform lesions were more frequent in malignant cases (29) compared to benign (9) with a significant p-value of 0.025. Midline appearance varied significantly with irregular (p = 0.003) and non-defined midlines being more common in malignant lesions. Junction characteristics showed high significance (p < 0.000) with interrupted and irregular junctions associated with malignancy. The presence of a bright edge was more common in malignant lesions (p = 0.044). Intracavitary fluid analysis indicated a significant difference (p = 0.021) with mixed fluid patterns seen mostly in malignant cases. The color score and vascular patterns were also highly significant (p < 0.000), with higher color scores and multiple vessel patterns linked to malignancy. Ultrasound diagnosis showed carcinoma endometrium and endometrial hyperplasia were predominantly malignant, while endometrial polyp was mostly benign (p < 0.000). Lastly, the mean endometrial thickness was significantly greater in malignant lesions (13.85 mm) compared to benign (9.19 mm) with a p-value of 0.000, indicating a strong correlation between increased thickness and malignancy (Table 3).

**Table 3:** Comparison of ultrasound features between benign and malignant lesions X^2 ^= chi-squared test; t = Student's t-test; LR = likelihood ratio.

		Type of lesion		Test value	P-value
		Benign	Malignant		
Uniformity	Non-uniform	9	29	X^2 ^= 5.032	0.025
	Uniform	7	5		
Midline appearance	Irregular	4	11	X^2 ^= 13.995	0.003
	Linear	7	1		
	Non-linear	2	6		
	Not defined	3	16		
Junction	Interrupted	0	10	X^2 ^= 38.390	0.000
	Irregular	0	6	LR = 46.113	0.000
	Linear	16	3		
	Not defined	0	12		
	Regular	0	3		
Bright edge	Absent	6	23	X^2 ^= 4.059	0.044
	Present	10	11		
Intracavitary fluid	Anechogenic	3	4	X^2 ^= 9.761	0.021
	Ground glass	1	7		
	Mixed	1	13		
	No fluid	11	10		
Color score	1	10	6	LR = 17.243	0.000
	2	4	6		
	3	2	10		
	4	0	12		
Vascular pattern	Circular vessel	1	1	LR = 41.012	0.000
	Multiple vessels (focal origin)	0	9		
	Multiple vessels (multiple focal origin)	0	13		
	No flow	9	3		
	Scattered vessels	1	5		
	Single vessel (with branching)	0	3		
	Single vessel (without branching)	5	0		
USG diagnosis	Carcinoma endometrium	0	11	X^2 ^= 22.475	0.000
	Endometrial hyperplasia	2	17		
	Endometrial polyp	14	6		
Endometrial thickness (mm), mean ± SD		9.19 ± 2.88	13.85 ± 4.466	t = 3.80	0.000

## Discussion

The findings of this study underscore the critical importance of the IETA scoring system in enhancing the diagnostic accuracy of ultrasound evaluations for AUB. By providing a standardized framework for assessing endometrial characteristics, IETA facilitates the differentiation between benign and malignant lesions, ultimately improving clinical decision-making processes for healthcare providers.

In the current study, it was identified that the mean endometrial thickness was significantly higher in malignant cases, averaging 13.85 ± 4.466 mm, compared to benign cases, which averaged 9.19 ± 2.88 mm. This difference was statistically significant, with a p-value of <0.001, indicating a strong association between increased endometrial thickness and the presence of malignancy. This finding is consistent with previous research conducted by Tabor et al., which reported that the median endometrial thickness observed in women diagnosed with endometrial cancer was significantly greater, measuring 3.7 times the thickness found in women without cancer at the same medical center. This comparison was made while controlling for menopausal status, ensuring that the difference in endometrial thickness was not influenced by variations in the menopausal state between the groups [8].

Similarly, Shokouhi reported findings that align with our study, noting that increased endometrial thickness was associated with endometrial hyperplasia and carcinoma, particularly in postmenopausal women [9]. These results underscore the relevance of endometrial thickness as a diagnostic parameter for identifying malignant endometrial conditions. In a study conducted by Heremans et al., the significance of endometrial thickness in differentiating benign from malignant lesions was further emphasized, with a higher mean thickness observed in malignant cases [6]. Several other studies have also reported similar findings, reinforcing the robustness of this diagnostic criterion [10,11].

The pattern of endometrial echogenicity was another critical parameter evaluated in our study. Non-uniform echogenicity was predominantly observed in malignant lesions, with heterogeneous patterns featuring irregular cysts being the most common. These findings align with the IETA consensus study conducted by Leone et al., which similarly noted that non-uniform echogenicity, especially with irregular cysts, is a significant indicator of malignancy [4,12,13]. Lin et al. also supported our findings, indicating that echogenicity patterns are crucial for differentiating endometrial pathologies. The presence of irregular midline appearances and interrupted myometrial junctions were significantly associated with malignancy in our study. These features were also highlighted by Leone et al. as critical markers for identifying malignant lesions [4]. Heremans et al. found that irregular and interrupted junctions are frequently observed in malignant cases, further reinforcing our findings [6].

In addition to these key parameters, this study highlighted the importance of a comprehensive ultrasound evaluation incorporating multiple IETA criteria to improve the accuracy of diagnosing endometrial pathologies. By combining measurements of endometrial thickness with assessments of echogenicity patterns and myometrial junction integrity, clinicians can more effectively differentiate between benign and malignant lesions, thereby improving patient outcomes through timely and accurate diagnosis.

It was also found that higher color scores and complex vascular patterns were significantly linked to malignant lesions. This finding highlights the critical role that vascular assessment plays in the evaluation of endometrial pathologies. Vascular patterns, particularly those with increased complexity and higher color Doppler scores, are indicative of malignancy, providing valuable diagnostic clues.

Lin et al.'s study supports our findings, demonstrating that combining IETA ultrasonographic characteristics with color Doppler scores improves the differentiation between benign and malignant lesions [5]. This approach allows for a more nuanced assessment by integrating multiple diagnostic parameters, thereby enhancing the accuracy of ultrasound evaluations. Similarly, multiple studies on IETA consensus emphasized the importance of vascular patterns and color scores in the comprehensive assessment of endometrial lesions [14-16]. Their findings corroborate our results, underscoring the significance of incorporating vascular characteristics into the diagnostic framework to distinguish between benign and malignant conditions effectively. By highlighting the association between higher color scores, complex vascular patterns, and malignancy, these studies collectively reinforce the utility of advanced ultrasonographic techniques in improving diagnostic precision for endometrial pathologies.

The integration of the IETA scoring system into routine ultrasound evaluations for AUB offers several clinical benefits and enhanced diagnostic accuracy by standardizing the assessment of endometrial characteristics. The IETA system improves the accuracy of distinguishing between benign and malignant lesions [17].

Accurate non-invasive diagnostic methods, such as advanced imaging and ultrasonography, reduce the need for invasive procedures like endometrial biopsy and hysteroscopy, thus minimizing patient discomfort and potential complications. It also offers several advantages over CT and MRI, including rapid scanning time, lack of radiation exposure, cost-effectiveness, and easy feasibility [18]. These methods enhance clinical decision-making by enabling standardized reporting, which allows for better risk assessment and personalized treatment strategies. Additionally, standardized systems ensure consistency in data collection and reporting, supporting research and long-term outcome monitoring [5,19]. This facilitates more accurate comparisons across studies and contributes to a stronger evidence base, ultimately improving patient care and advancing medical research in the management of endometrial pathologies.

While this study provides valuable insights, it is limited by its sample size and single-institution design. Future research with larger, multi-center cohorts is necessary to validate these findings and further refine the IETA scoring system. Additionally, incorporating advanced imaging techniques such as 3D ultrasound and MRI could enhance diagnostic accuracy and provide a more comprehensive evaluation of endometrial pathologies.

## Conclusions

The study highlights the critical role of the IETA scoring system in enhancing diagnostic accuracy for AUB through standardized ultrasound evaluations. The findings indicate that key parameters such as increased endometrial thickness, non-uniform echogenicity, irregular midline appearances, and complex vascular patterns are significantly associated with malignant lesions. Integrating IETA criteria, including comprehensive assessments of endometrial thickness, echogenicity patterns, and myometrial junction integrity, improves the differentiation between benign and malignant conditions. This approach supports more accurate, non-invasive diagnoses, reducing the need for invasive procedures and enabling better clinical decision-making and personalized treatment strategies.
